# Blood Pressure Control Is Associated with Moderate, but Not Necessarily High, Adherence to the DASH Diet in Older Adults

**DOI:** 10.3390/nu18020334

**Published:** 2026-01-20

**Authors:** Rafael Luengo-Dilla, Adriana Ortega-Hernández, Mónica Álvarez-González, Javier Gutiérrez-Corral, Javier Modrego, Macarena Torrego-Ellacuría, Sergio de la Torre-Rodríguez, Imane Jeidane-Bentefrit, Julia García-García, María Soledad Fragua-Gil, Dulcenombre Gómez-Garre, Arturo Corbatón-Anchuelo, The SEGOVIA Study Group

**Affiliations:** 1 Cardiovascular Risk and Microbiota Laboratory, Hospital Clínico San Carlos, Instituto de Investigación Sanitaria San Carlos (IdISSC), C/Prof. Martín Lagos, s/n, 28040 Madrid, Spain; rafael.luengo.externo@salud.madrid.org (R.L.-D.); adriana.ortega@salud.madrid.org (A.O.-H.); jgutierrezcorral@salud.madrid.org (J.G.-C.); javier.modrego@upm.es (J.M.); sergio.torre.externo@salud.madrid.org (S.d.l.T.-R.); ijeidane@ucm.es (I.J.-B.); 2 Centro de Salud La Sierra-Navafría (Segovia), Gerencia de Asistencia Sanitaria de Segovia, Paseo Conde de Sepúlveda, 1, 40002 Segovia, Spain; malvarezgon@saludcastillayleon.es; 3 Laboratory of Infectious Diseases, Hospital Clínico San Carlos-Instituto de Investigación Sanitaria San Carlos (IdISSC), C/Prof. Martín Lagos, s/n, 28040 Madrid, Spain; 4 ImFINE Research Group, Department of Health and Human Performance, Universidad Politécnica de Madrid (UPM), C/Martín Fierro, 7, 28040 Madrid, Spain; 5 Endocrinology of Metabolic Diseases Research Group, Hospital Clínico San Carlos, IdISSC, C/Prof. Martín Lagos, s/n, 28040 Madrid, Spain; macarena.torrego@salud.madrid.org; 6 Centro de Salud La Granja de San Ildefonso (Segovia), Gerencia de Asistencia Sanitaria de Segovia, Paseo Conde de Sepúlveda, 1, 40002 Segovia, Spain; jgarciagarc@saludcastillayleon.es; 7 Centro de Salud Carbonero El Mayor (Segovia), Gerencia de Asistencia Sanitaria de Segovia, Paseo Conde de Sepúlveda, 1, 40002 Segovia, Spain; mfragua@saludcastillayleon.es; 8 Physiology Department, Faculty of Medicine, Universidad Complutense de Madrid (UCM), Plaza Ramón y Cajal, s/n, 28040 Madrid, Spain; 9 Biomedical Research Networking Center in Cardiovascular Diseases (CIBERCV), Instituto de Salud Carlos III, Av. Monforte de Lemos, 3-5, 28029 Madrid, Spain; 10 Biomedical Research Networking Center in Diabetes and Associated Metabolic Disorders (CIBERDEM), Instituto de Salud Carlos III, Av. Monforte de Lemos, 3-5, 28029 Madrid, Spain

**Keywords:** DASH diet, hypertension, blood pressure control, older adults, Spain, observational study

## Abstract

Background/Objectives: Hypertension control remains a global challenge. Evidence on the association between adherence to the Dietary Approaches to Stop Hypertension (DASH) diet and blood pressure (BP) control in older Mediterranean populations is limited. We aimed to assess this association in Spanish older adults. Methods: This cross-sectional analysis included 371 participants (69 ± 9 years). Dietary intake was assessed using a validated 146-item food frequency questionnaire (FFQ), and DASH diet adherence was categorized as low, medium, or high. Multivariable logistic regression models were used to examine associations with BP control. Results: Among participants with hypertension (*n* = 218), 52.8% achieved adequate BP control and consumed significantly more low-fat dairy products (+56%) and less sodium (−11%) than those with uncontrolled BP. The low adherence group had lower proportion of participants with controlled BP (21%) than the medium and high adherence groups (36% and 39%, respectively) (*p* < 0.05). Across increasing DASH diet adherence categories, systolic blood pressure (SBP) and diastolic blood pressure (DBP) were 4–5 mmHg and 3–4 mmHg lower, respectively. Medium adherence to the DASH diet was independently associated with substantially lower odds of uncontrolled BP (OR = 0.37; 95% CI: 0.16–0.82; *p* = 0.015). High adherence showed a similar magnitude of association but did not reach statistical significance. Conclusions: In this cohort of older Spanish adults, moderate adherence to the DASH diet was associated with meaningful improvements in BP control, suggesting that achievable, intermediate levels of DASH diet adherence may be sufficient to improve hypertension management in real-world settings. Longitudinal studies are needed to confirm causality and long-term cardiovascular benefits.

## 1. Introduction

Hypertension is the leading modifiable risk factor for cardiovascular diseases and affects approximately 1.4 billion adults worldwide (about 33% of those aged 30–79 years). Despite available treatments, blood pressure (BP) control remains poor, with only around 23% of individuals achieving guideline-recommended targets, making hypertension a major cause of premature mortality [1].

In Spain, hypertension has a similar prevalence, affecting around 33% of adults and up to 70% of those aged over 60 years [2,3]. Although treatment rates are relatively high, approximately two-thirds of treated patients still fail to reach control targets [4]. This inadequate BP control is associated with more than 50% of cardiovascular deaths in individuals aged over 50 years and generates a substantial healthcare and economic burden [3,4,5,6].

The determinants of inadequate BP control are multifactorial and encompass suboptimal adherence to dietary recommendations, unhealthy lifestyles, presence of comorbidities, and lack of therapeutic adjustment [5,6]. In this context, lifestyle interventions, particularly dietary modifications, play a fundamental role in both the prevention and management of hypertension [7,8].

Among the most extensively studied dietary patterns for hypertension management, the Dietary Approaches to Stop Hypertension (DASH) diet stands out for its efficacy in reducing BP [9]. A systematic review and meta-analysis of 30 randomized controlled trials involving 5545 participants showed that, compared with control diets, DASH reduces both systolic blood pressure (SBP) and diastolic blood pressure (DBP), and this effect is even greater when combined with specific sodium restriction, demonstrating benefits at all levels of sodium reduction intake and proportional with baseline BP [9,10].

DASH dietary pattern is characterized by high consumption of fruits, vegetables, legumes, nuts, whole grains, and low-fat dairy products, alongside low intake of sodium, processed meats, and sugar-sweetened beverages [11]. Thus, it provides a high intake of potassium, magnesium, calcium, fiber, and antioxidant compounds [11]. Several physiological mechanisms may explain the association between DASH adherence and better BP control. Partial sodium reduction and higher intake of potassium, magnesium, and calcium may promote natriuresis, reduce arterial stiffness and peripheral vascular resistance, and improve endothelial function, leading to clinically relevant BP reductions [10,12,13,14]. Previous studies have shown that the DASH diet may improve insulin sensitivity, particularly in overweight and older adults, even in the absence of weight loss. In addition, higher DASH adherence has also been linked to lower levels of low-grade systemic inflammation, including reduced concentrations of C-reactive protein (CRP) and interleukin-6 (IL-6). These effects could be particularly relevant in older adults with salt sensitivity and endothelial dysfunction [15,16].

Numerous observational studies conducted in real-world settings have applied adherence scores to assess the relationship between compliance with the DASH diet and BP outcomes. Consistent findings indicate an inverse association between diet adherence and BP levels. Specifically, an observational analysis from the INTERMAP study reported that each 5-point increase in the DASH adherence score corresponds to an average reduction of approximately 1.35 mmHg in SBP [17].

Despite the demonstrated efficacy of the DASH diet in reducing BP, evidence on the association between adherence to the DASH dietary pattern and BP control as a clinical endpoint has received limited attention in older adults, particularly in Mediterranean or Southern European populations. Most studies have focused on short-term reductions in SBP and DBP and have been conducted in heterogeneous populations or non-Mediterranean settings [9,18]. Even in Mediterranean cohorts, available evidence has mainly addressed BP changes and cardiometabolic risk factors, while BP control in older adults has been less frequently examined [19]. Importantly, BP control, defined as achieving guideline-recommended targets, represents a distinct and clinically relevant endpoint beyond reductions in mean BP values and is emphasized in current hypertension guidelines because of its strong association with reduced cardiovascular morbidity and mortality [20]. In this context, evaluating the relationship between DASH diet adherence and BP control provides clinically meaningful information for hypertension management in routine practice. Therefore, the objective of this study was to analyse the association between adherence to the DASH dietary pattern and BP control in older Spanish adults participating in the SEGOVIA Study [21,22].

## 2. Materials and Methods

### 2.1. Study Design and Population

The SEGOVIA Study is a longitudinal, observational, community-based cohort study that recruited subjects aged 35 to 65 years between January 2000 and June 2003. The initial recruitment procedures, methodology, and selection criteria have been previously reported [21,22]. Briefly, participants were randomly selected using cluster sampling from the health census of the province of Segovia (Spain), representing a total target population of 63,417 individuals. Nine hundred participants constituted the initial cohort, with a mean age of 55 ± 12 years.

**Figure 1:**
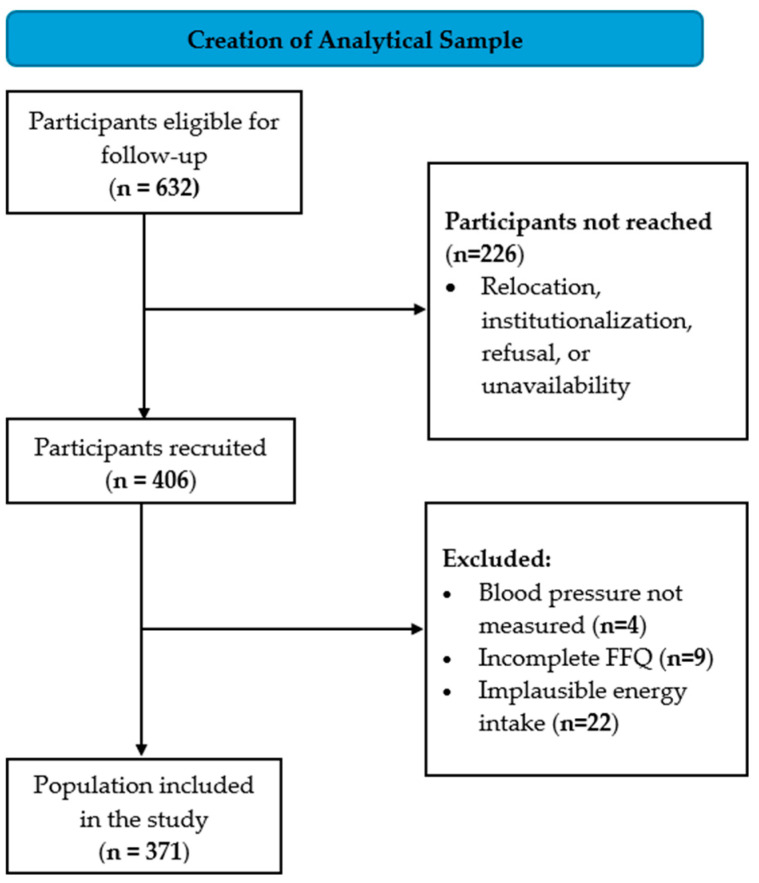
Flowchart of the study population. Abbreviations: FFQ, food frequency questionnaire.

A follow-up visit was conducted 20 years after recruitment (between 2021 and 2023), applying a cross-sectional design within this longitudinal cohort. Of the 632 participants eligible for follow-up, 226 were not reached, and 406 attended the follow-up visit. For the current study, we excluded participants with: (I) missing BP measurements (*n* = 4); (II) implausible energy intake (<600 or >5000 kcal/day) (*n* = 22); or (III) incomplete food frequency questionnaire (FFQ) (*n* = 9). Thus, 371 participants were included in the final analysis (Figure 1).

All participants provided written informed consent. The protocol was approved by the Ethics Committee for Research with Medicines of the Segovia Health Area, and the Ethics Committee of the Hospital Clínico San Carlos of the Health Service of the Community of Madrid (Nº CEIm: 19/409-E).

### 2.2. Assessment of Hypertension

Resting BP was measured with an automated BP cuff with the subject in a seated position. Three consecutive measurements were obtained at one-minute intervals. The final BP value was calculated as the mean of the second and third readings, in accordance with current clinical recommendations to minimize the impact of initial measurement variability [20].

Trained clinical personnel administered a standardized medical questionnaire to collect information on prior diagnosis of hypertension and current use of antihypertensive medications. Participants were classified as hypertensive if they met any of the following criteria: SBP ≥ 140 mmHg, DBP ≥ 90 mmHg, self-reported physician diagnosis of hypertension, or ongoing use of antihypertensive therapy [20]. BP control was defined as achieving SBP < 140 mmHg and DBP < 90 mmHg, as recommended by the 2022 Spanish Society of Hypertension–Spanish League for the Fight against Arterial Hypertension (SEH-LELHA) guidelines and used in epidemiological studies and routine clinical practice in Spain [20,23].

### 2.3. Nutritional Information and DASH Diet Score

Dietary intake was assessed using a validated 146-item semi-quantitative FFQ that was adapted from that used in the PREDIMED-Plus study for the Spanish population [24]. Average daily intake (g/day) was estimated by the Nutritional Epidemiology Unit of the University of Navarra [25]. As with all FFQ-based dietary assessments, recall bias and potential misclassification of nutrient intake (particularly sodium) are inherent limitations of this methodology [26,27].

Adherence to the DASH diet was evaluated using the Fung et al. [28] scoring method, which considers eight components: five considered healthy (fruits, vegetables, nuts and legumes, whole grains, and low-fat dairy) and three considered unhealthy (sodium, red and processed meats, and sugar-sweetened beverages). For each food group, consumption levels were divided into quintiles within the study population, stratified by sex. For healthy components, higher consumption quintiles received higher scores (5 points for the highest quintile, 1 point for the lowest). Conversely, for unhealthy components, lower consumption quintiles received higher scores (5 points for the lowest quintile, 1 point for the highest). The total DASH adherence score ranged from 8 to 40 points and was categorized as low (≤20), medium (21–28), or high (≥29), as previously reported [28]. These cut-off values have been widely used in epidemiological studies and have demonstrated validity in diverse populations, including older adults [29].

### 2.4. Sociodemographic, Clinical, and Anthropometric Data

Sociodemographic data, medical history, smoking status (never, former, current), and physical activity were collected through a structured medical questionnaire. Physical activity information included occupational activity, leisure-time exercise, sedentary time, and overall activity patterns, and was summarized as total weekly hours dedicated to physical activity (hours/week). Anthropometric measurements (weight, height, and waist circumference) were performed by trained clinical personnel following standardized protocols. Body mass index (BMI) was calculated by dividing weight (kg) by height squared (m^2^).

### 2.5. Statistical Analysis

Descriptive statistics were used to characterize demographic, clinical, and nutritional variables of study participants. Nutrient intakes were analysed both in absolute terms (grams, milligrams) and as percentages of total energy intake when appropriate. Normality of continuous variables was assessed using the Shapiro–Wilk test. Normally distributed variables are presented as mean ± standard deviation (SD), and group differences were evaluated with one-way ANOVA (or Welch’s ANOVA when variances were unequal). Non-normally distributed variables are expressed as median (interquartile range) and compared with the Kruskal–Wallis test. Categorical variables are reported as frequencies and percentages and tested using the chi-square test. Binary multivariable logistic regression models were applied to examine the association between adherence to the DASH diet and BP control, with low adherence as the reference group. Age, sex, BMI, and physical activity were included as covariates based on their established relationship with BP regulation. Antihypertensive treatment was not included as a covariate because this analytical approach was intentionally designed to reflect real-world BP control, were dietary behavior functions independently from pharmacological treatment, each as a distinct but complementary strategy. Additionally, no significant differences were observed between participants with controlled and uncontrolled BP, and adjusting for it could lead to overadjustment and attenuation of the associations of interest. Dietary variables and nutrient intakes were used to characterize DASH diet adherence and to describe dietary intake but were not included as independent variables in the regression models. Odds ratios (OR) with 95% confidence intervals (95% CI) were calculated for unadjusted and adjusted models. Missing data were handled using a complete-case approach. Of the 192 hypertensive participants available for the logistic regression analysis, 7 were excluded due to missing BMI values, resulting in a final analytical sample of 185 participants (96.4%). No imputation procedures were applied. As this was an observational analysis within an existing cohort, no a *priori* sample size calculation was performed. Post hoc power calculations for the fully adjusted logistic regression model (six predictors) indicated adequate statistical power (>0.80) to detect small-to-moderate effect sizes (f^2^ ≥ 0.08)**.** This analysis is intended to provide contextual support for understanding the study’s statistical capacity rather than as confirmatory evidence of statistical adequacy. All analyses were conducted using R software version 2025.09.1-401 (R Foundation for Statistical Computing, Vienna, Austria), with support from the Research Methodological Support Unit (UAMI). Statistical significance was defined as *p*-value < 0.05.

## 3. Results

### 3.1. Characteristics of the Study Population by Hypertension Status

A total of 371 participants (mean age 69 ± 9; 54.2% female) had complete dietary and BP data and were stratified into four groups according to BP status (Table 1). Overall, 218 (58.8%) participants had hypertension: 26 (11.9%) were newly diagnosed (de novo), while 192 (88.1%) had previously known hypertension, of whom 115 (59.9%) had controlled BP and 77 (40.1%) had uncontrolled BP. The remaining 153 (41.2%) participants were normotensive.

As shown in Table 1, participants with hypertension were significantly older and had higher BMI than normotensives, reaching obesity levels in the uncontrolled BP group. Similarly, waist circumference was significantly higher in both uncontrolled and controlled BP groups compared to the normotensive group. Fasting glucose levels were significantly elevated in both uncontrolled and controlled BP participants compared to normotensives. Regarding cardiovascular risk factors, the prevalence of type 2 diabetes mellitus (T2DM) was significantly higher in both uncontrolled and controlled BP groups compared to normotensives. In contrast, dyslipidemia prevalence did not differ across groups, despite significantly lower total cholesterol and higher triglycerides in participants with controlled and uncontrolled BP compared to normotensives. No significant differences in antihypertensive treatment patterns were observed between participants with controlled and uncontrolled BP.

### 3.2. Dietary Intake and Key Nutritional Components

Dietary intake differed significantly across BP control groups. As shown in Table 2, participants with uncontrolled BP reported significantly higher total energy intake compared to those with controlled BP, while *de novo* and normotensive participants showed intermediate values.

The percentage of calories from protein was significantly higher in the controlled BP group compared to the other three groups. Sodium intake was approximately 11% higher in the uncontrolled BP group compared to the controlled BP group, while no significant differences were observed among the other groups. The controlled BP group also consumed approximately 56% more low-fat dairy products compared to *de novo* hypertensive individuals and 9% more compared to normotensive participants. No significant differences were found across BP status groups for other nutrients (Table 2).

### 3.3. BP Across DASH Diet Adherence Levels

For the analysis of DASH diet adherence in relation to BP control, participants with newly diagnosed (*de novo*) hypertension were excluded to maintain methodological rigor. The remaining 345 participants were distributed as follows: 78 (23%) with low adherence, 211 (61%) with medium adherence, and 56 (16%) with high adherence.

**Figure 2:**
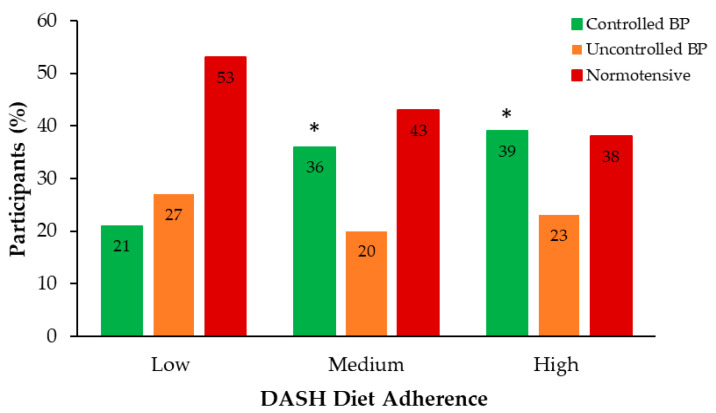
Distribution of BP status groups across DASH diet adherence levels. Sample size (*n*): Low = 78; Medium = 211; High = 56. * *p* < 0.05 vs. Low adherence. Abbreviations: BP, blood pressure; DASH, Dietary Approaches to Stop Hypertension.

Analysis of BP across adherence levels revealed a distinct pattern in BP control status (Figure 2). The low adherence group had a significantly lower proportion of participants with controlled BP (21%) compared to the medium and high adherence groups (36% and 39%, respectively) (*p* < 0.05).

**Figure 3:**
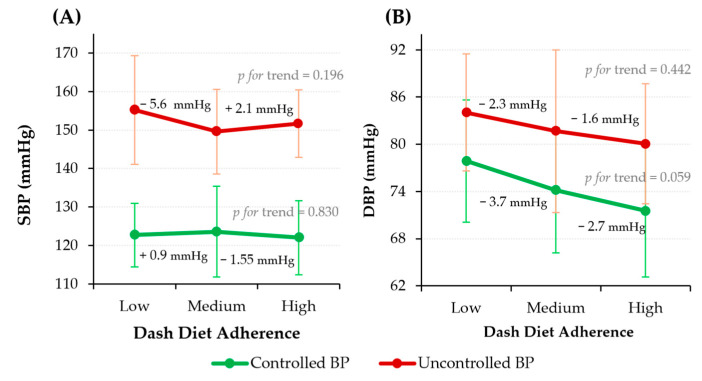
SBP (**A**) and DBP (**B**) across DASH diet adherence levels stratified by BP control status. Data are presented as mean ± SD. Sample size (*n*): Low = 78; Medium = 211; High = 56. Abbreviations: BP, blood pressure; DBP, diastolic blood pressure; SBP, systolic blood pressure.

As shown in Figure 3, SBP and DBP tended to decrease across DASH diet adherence categories. For SBP, the controlled BP group did not show significant changes, whereas participants with uncontrolled BP and low adherence showed higher values than those with medium and high adherence. In contrast, DBP exhibited a more consistent declining pattern with increasing adherence in both groups. Among individuals with controlled BP, DBP decreased progressively across adherence levels, approaching statistical significance (*p* = 0.059). Similarly, participants with uncontrolled BP exhibited a decreasing numerical trend across low, medium, and high adherence categories. Although the observed differences in BP across DASH diet adherence categories were modest, evidence from large epidemiological studies indicates that even small reductions in SBP and DBP are associated with meaningful reductions in cardiovascular risk at the population level [30].

As shown in Table 3, participants with low adherence to the DASH diet exhibited significantly higher BMI compared to those with medium and high adherence. Smoking prevalence decreased significantly across adherence categories. No significant differences were observed across groups regarding the prevalence of T2DM, dyslipidemia, or physical activity levels. However, a descending trend was observed for waist circumference, SBP and DBP, total cholesterol, LDL cholesterol, and triglycerides, with comparable values between medium and high adherence groups.

### 3.4. Logistic Regression on BP Control

Multivariable logistic regression models were constructed to examine the association between DASH diet adherence and BP control (Table 4). Medium adherence to the DASH diet was consistently associated with lower odds of uncontrolled BP across all models. In the fully adjusted model (Model 6), participants with medium adherence had 63% lower odds of uncontrolled BP compared with the reference group (low adherence) (OR = 0.37; 95% CI: 0.16–0.82; *p* = 0.015).

High adherence showed a similar reduction in the odds of uncontrolled BP. In the fully adjusted model, high adherence to the DASH diet showed a trend toward better BP control, although the association did not reach conventional statistical significance (Table 4). Notably, the near-identical point estimates for medium and high adherence suggest that achieving at least moderate adherence may be sufficient to obtain substantial clinical benefits in BP control.

## 4. Discussion

In this follow-up study of Spanish adults from the province of Segovia aged 55 years or older, hypertension was highly prevalent, affecting almost 60% of participants, of whom 40.1% remained uncontrolled despite treatment. The main finding of our study is that medium adherence to the DASH diet was independently associated with lower odds of uncontrolled BP, suggesting that dietary behavior may contribute to hypertension management in older adults living in Mediterranean settings. To our knowledge, this is among the first studies to assess the relationship between DASH diet adherence and BP control in an older Spanish cohort, contributing evidence to Mediterranean settings.

Hypertension remains the leading modifiable risk factor for death and disability worldwide [2]. In Spain, despite improvements in its management over time, BP control remains suboptimal [3,31]. In our study, the prevalence of uncontrolled BP among individuals with hypertension was comparable to that reported in the ENRICA study, a large population-based survey conducted in Spain in 2011, where approximately 52% of treated patients with hypertension did not achieve adequate BP control [32]. More recently, in 2019, it was estimated that approximately 43% of adults aged 30–79 years remain uncontrolled despite treatment [3]. Similar data have also been reported in the European Health Examination Survey, where 37–45% of treated hypertensive adults across Europe remained uncontrolled [33]. The concordance between these epidemiological studies and our findings, despite differences in study populations, measurement protocols, and healthcare contexts, suggests that the challenge of achieving adequate BP control persists, even in the context of improved pharmacological therapy and healthcare access.

Randomized controlled trials have demonstrated that adherence to the DASH diet induces substantial reductions in BP, particularly when combined with other lifestyle measures such as weight control [34,35]. Furthermore, a recent meta-analysis of 30 randomized controlled trials reported mean reductions of 3.2 mmHg in SBP and 2.5 mmHg in DBP associated with DASH diet interventions, in both hypertensive and normotensive individuals [9]. However, these controlled trials typically involve intensive, multifaceted interventions and motivated participants, conditions that rarely occur in routine clinical practice. Observational studies have reported more modest but significant reductions in SBP (SMD = −0.18; 95% CI: −0.32 to −0.04) and DBP (SMD = −0.13; 95% CI: −0.19 to −0.06) associated with greater adherence to the DASH diet [36]. In our study, SBP and DBP were 4–5 mmHg and 3–4 mmHg lower, respectively, among participants with medium and high adherence to the DASH diet, compared with those with low adherence, demonstrating that observational associations between DASH adherence and BP control are also present in an older population, an age group often underrepresented in dietary intervention trials. Importantly, even modest reductions in BP could be clinically relevant. Epidemiological studies have reported that a reduction of approximately 5 mmHg in SBP is associated with a ~10% reduction in major cardiovascular events, whereas a 2 mmHg decrease in DBP may reduce the risk of coronary heart disease and stroke by 6–15% [37]. In our study, medium adherence to the DASH diet was independently associated with a reduction in the odds of uncontrolled BP. Consistent with our results, the PREDIMED-Plus study reported that greater adherence to the DASH diet was longitudinally associated with significant reductions in SBP (−0.57 mmHg) and DBP (−0.15 mmHg), as well as improvements in multiple cardiometabolic risk factors in a high cardiovascular risk cohort [19]. However, as most real-world evidence is cross-sectional, longitudinal studies are needed to confirm the persistence, causality, and long-term impact of these associations on BP and cardiovascular outcomes.

Our findings indicate that moderate adherence to the DASH diet may be sufficient to achieve BP control, suggesting a possible threshold effect. These results differ from those reported by Chefran et al. [38] in a French cohort of treated adults with hypertension, who reported that low or medium adherence to general dietary recommendations was associated with a higher probability of uncontrolled BP compared with high adherence. Methodological differences between the studies, including age, population characteristics, and the cut-off points used, may explain these discrepancies. Moreover, higher DASH scores have been associated with lower cardiovascular mortality in patients with hypertension, indicating that even partial adherence may confer significant benefits [39]. In our study, the lack of statistical significance in the high-adherence group likely reflects limited statistical power rather than an absence of effect. From a mechanistic perspective, the DASH diet influences BP through multiple complementary pathways, and partial dietary changes may be sufficient to induce meaningful improvements in fluid balance, peripheral vascular resistance, and endothelial function. Overall, these results support moderate DASH diet adherence as a realistic and effective target for BP control in older adults, while underscoring the need for larger studies to clarify the additional benefits of higher adherence.

Recent studies have demonstrated that BP control depends on multiple lifestyle factors beyond diet alone. For instance, Worku et al. [40] reported that 66.2% of patients attending public hospitals in Addis Ababa had uncontrolled hypertension, with poor dietary adherence, excess body weight, physical inactivity, and low medication compliance, all contributing to poorer outcomes. Consistent with these findings, in our study, participants with low DASH diet adherence showed significantly higher BMI and a greater prevalence of smoking, two major determinants of high BP and cardiovascular risk. This clustering of risk factors underscores the complexity of hypertension management in older adults and highlights the need for comprehensive, yet realistic, lifestyle interventions. In this regard, high adherence to strict diets may be difficult to sustain in older adults; therefore, gradual interventions, such as increasing consumption of fruits, vegetables, and low-fat dairy while progressively reducing sodium intake, may be more acceptable and sustainable than strict dietary prescriptions. From a clinical and public health perspective, these findings are highly relevant in real-world settings, where strict adherence to the DASH diet is difficult to achieve, particularly among older Spanish adults due to entrenched dietary habits, cultural preferences, comorbidities, and social factors. Observational studies indicate that less than 25–30% of older adults reach high DASH diet adherence, even in motivated populations [19,35]. Therefore, our observation that moderate rather than high adherence is associated with better BP control underscores intermediate dietary changes as a more realistic and achievable clinical target.

This study has several strengths. First, we used a population-based cohort of older adults, which provides real-world evidence on dietary patterns and BP control in a vulnerable age group. Second, the DASH score was calculated using a validated FFQ adapted to the Spanish population. Third, we included multivariable models adjusted for key lifestyle and anthropometric factors, strengthening the validity of our findings.

However, several limitations should be acknowledged. First, the cross-sectional design does not permit establishing causal relationships. Reverse causality is possible; participants with better BP control may be more likely to adhere to a healthy diet. Second, dietary assessment using FFQ is subject to inherent limitations, including recall bias, potential misclassification of sodium intake, and underestimation of actual food intake. Third, BP was measured during a single study visit, which may not fully reflect usual BP levels and may be influenced by white-coat or masked hypertension. While current guidelines recommend repeated measurements for diagnosis and classification [20], our approach was supported by clinical history, antihypertensive treatment, and regular follow-up by primary care physicians. Ambulatory or home BP monitoring data were not available, though requesting home measurements could have introduced additional measurement bias due to untrained self-measurement. Additionally, the timing of antihypertensive medication intake relative to BP measurement was not systematically recorded. Fourth, medication adherence, an important determinant of BP control, was not objectively monitored. Therefore, residual confounding cannot be excluded, as differences in adherence to antihypertensive treatment may have influenced BP control independently of dietary habits. Future studies should incorporate direct measures of medication adherence (e.g., pill counts, pharmacy records). Finally, the study’s restriction to older adults from a single Spanish province (Segovia) limits generalizability to younger populations and other regions, particularly non-Mediterranean areas.

## 5. Conclusions

In conclusion, medium adherence to the DASH diet was independently associated with a lower likelihood of uncontrolled BP in Spanish older adults from the SEGOVIA Study. These findings suggest that moderate DASH diet adherence may be clinically significant for improving BP control in aging Mediterranean populations without the need for strict dietary compliance. Extrapolation to younger adults or non-Mediterranean populations should be made with caution. Further longitudinal studies are warranted to clarify temporal relationships and to assess the long-term implications of DASH diet adherence for cardiovascular outcomes.

## Figures and Tables

**Table 1:** Descriptive analysis of the clinical variables of the participants by BP status.

	Uncontrolled BP (*n* = 77)	Controlled BP (*n* = 115)	*De novo* Hypertension (*n* = 26)	Normotensive (*n* = 153)
**Age (years)**	72 ± 10 ^c^	71 ± 9 ^c^	72 ± 9 ^c^	66 ± 8
**Women, *n* (%)**	41 (53%)	65 (57%)	14 (54%)	81 (53%)
**BMI (kg/m^2^)**	30 ± 5 ^c^	29 ± 4 ^c^	31 ± 4 ^c^	27 ± 4
**Waist circumference (cm)**	101 ± 13 ^c^	100 ± 13 ^c^	98 ± 11	93 ± 13
**SBP (mmHg)**	151 ± 12 ^a,c^	123 ± 11 ^b,c^	148 ± 8 ^c^	118 ± 12
**DBP (mmHg)**	82 ± 9 ^a,c^	74 ± 8 ^b^	80 ± 8 ^c^	74 ± 8
**T2DM (%)**	30 (39%) ^c^	37 (32%) ^c^	7 (27%)	18 (12%)
**Dyslipidemia, *n* (%)**	67 (87%)	99 (86%)	21 (81%)	123 (80%)
**Current smoker, *n* (%)**	4 (5%)	7 (6%)	1 (4%)	21 (14%)
**PA (hours/week)**	7 ± 7	6 ± 6	7 ± 6	6 ± 6
**Fasting glucose (mg/dL)**	108 ± 27 ^c^	105 ± 21 ^c^	102 ± 18	96 ± 14
**Total cholesterol (mg/dL)**	196 ± 35 ^c^	188 ± 38 ^c^	202 ± 33	208 ± 36
**LDL-C (mg/dL)**	119 ± 32	111 ± 35 ^c^	124 ± 28	128 ± 31
**HDL-C (mg/dL)**	56 ± 14	56 ± 13 ^c^	54 ± 10	61 ± 16
**Triglycerides (mg/dL)**	105 ± 46 ^c^	111 ± 60 ^c^	110 ± 55	94 ± 46
**Treatment:**				
**ACEi/ARB, *n* (%)**	55 (71%)	75 (65%)	—	—
**CCB, *n* (%)**	6 (8%)	14 (12%)	—	—
**Beta-blockers, *n* (%)**	7 (9%)	17 (15%)	—	—
**Diuretics, *n* (%)**	24 (31%)	42 (36%)	—	—
**Others, *n* (%)**	4 (5%)	7 (6%)	—	—

Data are presented as mean ± SD for continuous variables and as *n* (%) for categorical variables. Superscript letters indicate statistically significant differences between groups (*p* < 0.05): ^a^ differs from the controlled BP group, ^b^ differs from the *de novo* Hypertension group and ^c^ differs from the normotensive group. Abbreviations: BP, blood pressure; BMI, body mass index; SBP, systolic blood pressure; DBP, diastolic blood pressure; T2DM, type 2 diabetes mellitus; PA, physical activity; LDL-C, low-density lipoprotein cholesterol; HDL-C, high-density lipoprotein cholesterol; ACEi, angiotensin-converting enzyme inhibitor; ARB, angiotensin II receptor blocker; CCB, Calcium channel blockers.

**Table 2:** Descriptive analysis of nutritional variables of the participants by BP status.

	Uncontrolled BP (*n* = 77)	Controlled BP (*n* = 115)	*De novo* Hypertension (*n* = 26)	Normotensive (*n* = 153)
**Total energy (kcal)**	2549 [2117,3470] ^a^	2286 [1892,2723]	2584 [1845,3250]	2444 [1939,3151]
**Protein (%)**	18 ± 3 ^a^	19 ± 4 ^b,c^	16 ± 3	17 ± 4
**Carbohydrates (%)**	44 ± 8	42 ± 8	46 ± 8	42 ± 8
**SFA (%)**	10 [9,12]	10 [8,12]	10 [8,11]	11 [9,12]
**Alcohol (g)**	2 [0,12]	1 [0,9]	1 [0,8]	3 [1,10]
**Total fiber (g)**	33 [25,30]	28 [21,31]	30 [23,32]	30 [22,32]
**Cholesterol (mg)**	491 [366,583]	445 [342,566]	377 [279,608]	471 [379,619]
**Magnesium (mg)**	475 [374,569]	418 [336,521]	418 [339,589]	431 [338,548]
**Calcium (mg)**	1138 [936,1402]	1105 [831,1425]	1000 [613,1355]	1064 [800,1388]
**Potassium (mg)**	5406 ± 1540	5050 ± 1828	4940 ± 1609	5072 ± 1753
**Sodium (mg)**	2683 [2010,3574] ^a^	2407 [1747,2909]	2401 [1541,3155]	2484 [1971,3252]
**Vegetables (g)**	342 [229,466]	305 [209,438]	278 [185,386]	309 [206,419]
**Fruits (g)**	386 [259,604]	370 [253,555]	405 [253,578]	390 [243,568]
**Whole grains (g)**	0 [0,15]	0 [0,23]	0 [0,26]	0 [0,24]
**Low-fat dairy (g)**	200 [125,500]	218 [200,500] ^b,c^	140 [0,298]	200 [52,325]
**Legumes (g)**	38 [27,59]	40 [25,62]	46 [25,73]	37 [25,55]
**SSB (g)**	0 [0,27]	13 [0,28]	13 [0,77]	13 [0,56]
**Red/processed meat (g)**	89 [60,150]	87 [55,123]	70 [49,98]	87 [57,143]

Data are presented as mean ±SD for normally distributed continuous variables and as median [interquartile range] for non-normally distributed continuous variables. Superscript letters indicate statistically significant differences between groups (*p* < 0.05): ^a^ differs from the controlled BP group, ^b^ differs from the *de novo* Hypertension group and ^c^ differs from the normotensive group. Abbreviations: BP, blood pressure; SFA, saturated fatty acids; SSB, sugar-sweetened beverages.

**Table 3:** Descriptive analysis of clinical variables by level of adherence to DASH diet.

	Low Adherence	Medium Adherence	High Adherence
	(Score ≤ 20) (*n* = 78)	(Score 21–28) (*n* = 211)	(Score ≥ 29) (*n* = 56)
**Age (years)**	69 ± 9	69 ± 9	71 ± 10
**Women, *n* (%)**	41 (53%)	119 (56%)	27 (48%)
**BMI (kg/m^2^)**	29 ± 4 ^a,b^	28 ± 5	28 ± 4
**Waist circumference (cm)**	100 ± 12	96 ± 14	96 ± 14
**SBP (mmHg)**	131 ± 18	126 ± 17	127 ± 17
**DBP (mmHg)**	78 ± 9	75 ± 9	74 ± 9
**T2DM (%)**	18 (23%)	50 (24%)	17 (30%)
**Dyslipidemia, *n* (%)**	66 (85%)	178 (84%)	45 (80%)
**Current smoker, *n* (%)**	14 (18%) ^a,b^	18 (9%) ^b^	0 (0%)
**PA (hours/week)**	5 ± 6	7 ± 6	7 ± 6
**Fasting glucose (mg/dL)**	101 ± 15	101 ± 19	107 ± 30
**Total cholesterol (mg/dL)**	204 ± 34	198 ± 37	193 ± 42
**LDL-C (mg/dL)**	124 ± 31	120 ± 33	114 ± 36
**HDL-C (mg/dL)**	58 ± 17	59 ± 14	58 ± 14
**Triglycerides (mg/dL)**	112 ± 64	98 ± 46	103 ± 50

Data are presented as mean ± SD for continuous variables and as *n* (%) for categorical variables. Superscript letters indicate statistically significant differences between groups (*p* < 0.05): ^a^ differs from medium adherence and ^b^ differs from high adherence. Abbreviations:; BMI, body mass index; SBP, systolic blood pressure; DBP, diastolic blood pressure; T2DM, type 2 diabetes mellitus; PA, physical activity; LDL-C, low-density lipoprotein cholesterol; HDL-C, high-density lipoprotein cholesterol.

**Table 4:** Association between DASH diet adherence categories and BP control in binary logistic regression models.

	Medium Adherence		High Adherence	
	(Score 21–28) (*n* = 115)		(Score ≥ 29) (*n* = 34)	
Model	OR (95% CI)	***p***-Value	OR (95% CI)	***p***-Value
**Model 1:** Unadjusted	0.43 (0.20–0.90)	0.026	0.45 (0.17–1.15)	0.098
**Model 2:** Adjusted for age	0.40 (0.19–0.85)	0.019	0.40 (0.15–1.05)	0.066
**Model 3:** Adjusted for sex	0.43 (0.20–0.90)	0.026	0.45 (0.17–1.14)	0.095
**Model 4:** Adjusted for physical activity	0.41 (0.19–0.88)	0.023	0.44 (0.17–1.13)	0.091
**Model 5:** Adjusted for BMI	0.41 (0.19–0.89)	0.025	0.44 (0.16–1.15)	0.098
**Model 6:** Adjusted for age, sex, physical activity, and BMI	0.37 (0.16–0.82)	0.015	0.37 (0.13–1.01)	0.055

Values are odds ratios with 95% confidence intervals estimated from binary logistic regression models. Reference category: low DASH diet adherence (≤20). Abbreviations: BP, blood pressure; BMI, body mass index; CI, confidence interval; OR, odds ratio.

## Data Availability

The data supporting the findings of this observational study are available from the corresponding authors upon reasonable request. The data are not publicly available due to privacy and ethical restrictions.

## Abbreviations

The following abbreviations are used in this manuscript:

BP	Blood pressure
CI	Confidence interval
DASH	Dietary Approaches to Stop Hypertension
SBP	Systolic blood pressure
DBP	Diastolic blood pressure
FFQ	Food frequency questionnaire
BMI	Body mass Index
SD	Standard Deviation
OR	Odds Ratio
T2DM	Type 2 Diabetes Mellitus

## Appendix A

The SEGOVIA Study Group as of 2021–2023: CENTRO DE SALUD CANTALEJO: Carmen Sánchez Peinador, Rosa Amparo Ramos Herranz, Teresa Montero Carretero, Magdalena Garrido Mesa, Delia de Lucas San Atanasio, Begoña Torijos Arévalo, Henar Pascual Lázaro, Ana María De Lucas Herrero, María Teresa Sánchez García, Laura Del Río Arranz, Adrián González Pérez, Noelia De La Esperanza Esteban, Rocío Pascual Arévalo. CENTRO DE SALUD CARBONERO EL MAYOR: María Soledad Fragua Gil, Lucía Corral Cuevas, María Jesús Blanco Ledesma, Alfonso de Santos López, María Concepción Manrique de la Fuente, María Ángeles Lazcoz Fontal, María Teresa Jiménez López, Virginia Silva Guisalola, Elena Muñoz Alonso, Monserrat Fuencisla Gil Martín, José Antonio Franco Yagúe, Vanessa Gallardo Ficorilli, Connie Pierina Gallardo Ficorilli, Armen Yesayan Karapetyan, María Areños Camarero, Gregoria Manso Fernández, Cristina de la Cruz Maeso, María Espíritu Santo Otero Herrero, Carmen Tapia Valero, María Mercedes Herranz Rosa, Carmen González Ferreiro, María Jesús Cardiel Mañas, Javier Velasco Redondo, María Teresa Llorente Gonzáles, Yolanda Escribano Herranz, Natalia Gómez Muñoz, Patricia Redondo Arranz, Raquel Maroto Gómez, Rosa María Rodrigo de Frutos, Rosa Álvarez Valverde, Juan Antonio Hidalgo Pardos, María Belén Diaz-Pavón Romo. CENTRO DE SALUD CUÉLLAR: Marta León Espinosa, Yleison Rodríguez Rodríguez, María Victoria Martín Pérez, Martín Riofrío Pastor, Mónica Pilar Ramírez Gordillo, José Manuel Vicente Muñoz, José Antonio Moro Santirso, Raquel Lozano Gimón, Laura Frutos de la Rosa, Nuria de Frutos Escobar, María Belén Sánchez Martín, María García Lozano, Eva María García García, María Yolanda Escribano Herranz, Beatriz Díaz Navarro, María Montserrat Cuéllar Lázaro, María Rodríguez San Bruno, Ana Martín Rodríguez. CENTRO DE SALUD DE EL ESPINAR: Laura Cortés García, María Luisa López García de Paredes, Ana Delia Martín Almeida, Cristina Martín Tapia, Pavel Pirogov, Alba María Rodríguez Maya, Lidyse Hernández Hernández, Olena Kushnirenko Bezugla, Daniela Andreina Rodríguez Arrieta, Mercedes Arranz Valentín, María Luisa de la Calle Ayuso, José María García Portero, Ferda Gazi, Juan Pedro Haba Gómez, Miroslawa Osiecka Stalexska, Estrella Tardón Santos, Jesús Alberto Valdaliso González, Nieves Blanco Callejo, María Inmaculada Alonso, Alba de Santos, María Pilar Guerra Andrades, Andrea Lázaro Parra. CENTRO DE SALUD NAVA DE LA ASUNCIÓN: Cathy Soto Barzola, Rosa García López-Tello, Juan Carlos Olivo Takano, Rafael Sánchez Díaz, Cecilia Edineth Camero, Alberto González Bazán, Adoración Pascual Matesanz, Clementina Gamarra González, Ana Katyuhuska Chung Zafra, Raisa Álvarez Paniagua, Justo Sebastián López, María Amaya Hernández Rubio, Arancha Jiménez Maroto, Héctor Aceves Gamarra, María Montero Sanz. Enfermeras: Vanesa Calle Prieto, Mª Teresa Arranz Matesanz, José Joaquín Vélez López, Rocío Alonso Pérez, Inés Domínguez Martín, Mª Estrella Santos Rubio, Encarnación Martín de Pedro, María López Seisdedos, Esther González López, Cristina Sáez García. CENTRO DE SALUD NAVAFRÍA: Irene Noa Carrión, Jesús Izquierdo Sánchez, Julia Reques Sebastián, Mónica Álvarez González, Nuria de Andrés Antón, María del Socorro García Tomé, Raquel Yustas Domínguez, Eva García Arahuetes. CENTRO DE SALUD RIAZA: Santiago Jiménez García, Concepción Kurtz Luna, Carlos David Pérez Gómez, Cesar Augusto Castaño Rodríguez, Marta Arribas Domínguez, José Montoro Caballero, Josefina Sánchez-Monge Domínguez, José Luis Alcalde San Miguel, Rafael Bladimir Hernández Zabala. Susana de Diego García, Eva María Álvarez de Castro, Bienvenida Machado Simal, Carmen Muñoz Arias, Javier Arnao Rodríguez, Patricia Villoria García, María Eugenia Barreno de Pablos, Alba de Santos Carrión, Inmaculada Alonso Niño. CENTRO DE SALUD SACRAMENIA: Ramiro Santaolaya García, Juan Carlos Rodríguez Mayo, María Jesús Pinilla Sánchez, Antonio de Pablo Bachiller, Diego Francisco Barrionuevo Burgos, Fuencisla Quintana Rojo, Raúl Santos González, José María Murciego Zarzosa, María Yolanda Mancebo Rojo, Manuel Aceves Gamarra, Eva María Herrero Callejo. CENTRO DE SALUD DE SAN ILDEFONSO: Julia García García, María Antonia Casado Velázquez, Lehdia Mohamed Dafa, Mª del Mar Viribay Montilla, Luis Alfonso Santos. Adriana Valdenebro Hernández, Jesús Sierra García, Josefina Coto Sanz, Noelia Herrero López, Mª de la Palma González Manso. CENTRO DE SALUD SEGOVIA RURAL: María Dolores Piñuela de la Calle, Teresa Arranz Olmos, Enrique Arrieta Antón, Yolanda Artiñano Del Pozo, Carlos Avellaneda Martínez, Euro Jesús Barrios Guedez, Pinar Benito Zamarriego, María De La Calle Del Barrio, Javier González Padilla, Marianny Del Carmen Guzmán Jumelles, Ana Isabel Herrero Cecilia, Esther Herrero Rincón, Olena Kushnirenko Bezugla, Gregoria Manso Fernández, María Regidor Miguel, María Antonia Sánchez García, Francisco Sánchez Juan, Catherine Paola Soto Barzola, Mª Mar Aguirre López-Aberasturi, Pinar Calvo de Lucas, Oscar García Alonso, María Martín Manso, Carmen Melero Sacristán, Carmen Menéndez Cuenca, M Jesús Merino Lucía, Carmen Santiago Trapero, Montserrat Sanz García, M Cruz Velasco Gil, M Henar Velasco Gutiérrez, Félix Yubero Martin, Virginia Goya Pérez, Cristina de Prado Casado, Beatriz Herranz de Pablos, Miriam Velasco Manso, Cristina Sacristán Alonso, Belinda Hernando Higuera, David Velasco del Olmo, Jorge Ferrete Sánchez, Olalla Millán García. CENTRO DE SALUD SEGOVIA I: Benito de la Hoz García, Virginia Nieto Lorasque, Agustín Moreno Aragoneses, Mercedes de Santos Román, Inmaculada López Rodríguez, Alicia Barrero Nadales, Corina Torres Barriga, Verónica Huacón Mendoza, Ana I. Cordero Sanz, Maite Herranz Olmos, Cristina Velarde Mayol, Raquel Cristóbal de la Cruz, Raquel Cristóbal de la Cruz, Ana I. Rivera Blanco, Susana Abad Arribas, Fuencisla Díez Fernández, Ana Gloria Álvarez Reino, Mª Paz Gómez Garcimartín, Emma Álvarez Martín, Cristina Sánchez Arenas, Gema Palacios Manso, Mª Ángeles Delgado Fraile, Eva Merinero san Miguel, Eduardo Gil González, Carolina Marcos Barreno. CENTRO DE SALUD SEGOVIA II: Lucía Fuentes Fuentes, Juan Manuel de Andrés Rubio, Yolanda Calleja Gordaliza, María José de Andrés Francés, Esther Herrero Rincón, Juana Alonso Barbolla, Rucadén Pérez Toledo, Julia García García, Beatriz Herranz De Pablos, Elena García María, Elvira Mª Otero Diéguez, María Serrano Redondo, Mª Rosario Victoria Romo, Carmen Cuellar Herranz, Miriam Sanz Cristóbal, Mª Ángeles Requero, Juana Lucia Lázaro Valverde. CENTRO DE SALUD SEGOVIA III: Manuel Hernández Machado, Mónica Herranz Garriga, José Rodríguez Sanz, María Tana Martínez Vilanova, Ana María Guío López, Juan Francisco Gil García, Ricardo Borges Oliveira, Pedro Pascual Alonso, Leticia Silvia Iglesias, Mª Henar Álvarez Santos, Mª Zulema Cabrero Carrera, Aurora de la Flor Pérez, Carlos Garrote Tapia, Teresa López Fernández Quesada, Montserrat Martín Bartolomé, Adriana Peña de Allas, Paula Martín Álvarez, Elena Sacristán Hernández, Leila Robaina Yanez, María Serrano Redondo, Marta Gómez Ruíz. CENTRO DE SALUD DE SEPÚLVEDA: Rosa María Fernández Santa Teresa, Encarnación Gaitero Rodríguez, Luis Sebastián González Trigueros, Sara Matesanz García, Martín Merino Segovia, Plinio Abel Montes de Oca Jiménez, Estrella Barrio Garrido, Luliana Botas, Mª Mar Coto Sanz, Sonia Fraguas Martínez, María Ángeles Ruíz Bobillo. CENTRO DE SALUD DE VILLACASTÍN: Cristina Crespo González, Cinthya Paola Guachan Salazar, Mª Teresa López Nogales, María Marcos Hidalgo, Ana Palacios Herrero, Javier Velasco Redondo, Concepción Vicente Cuadrado. HOSPITAL CLÍNICO SAN CARLOS: David Romero-Gómez, Zaira García-Blázquez, María Teresa Martínez-Larrad.
